# Supplementary material for: Deep scanning lysine metabolism in Escherichia coli
Source: Mol Syst Biol. 2018 Nov 26;14(11):e8371. doi: 10.15252/msb.20188371 (PMC6254735; doi:10.15252/msb.20188371)
Supplement: Supplementary file 1 — Appendix [file MSB-14-e8371-s001.pdf]

Deep scanning lysine metabolism in *Escherichia coli*

Marcelo C. Bassalo, Andrew D. Garst, Alaksh Choudhury, William C. Grau, Eun J. Oh, Eileen Spindler, Tanya Lipscomb, Ryan T. Gill

APPENDIX FIGURES

Table of Contents

Figure S1. Histogram of in the selected vs no selection sample...............................2

Figure S2. Fraction of lysP mutants in the population...3

Figure S3. Expression and purification of DapF variants. 4

Figure S4. Example of the kinetic assay performed for DapF..................................5

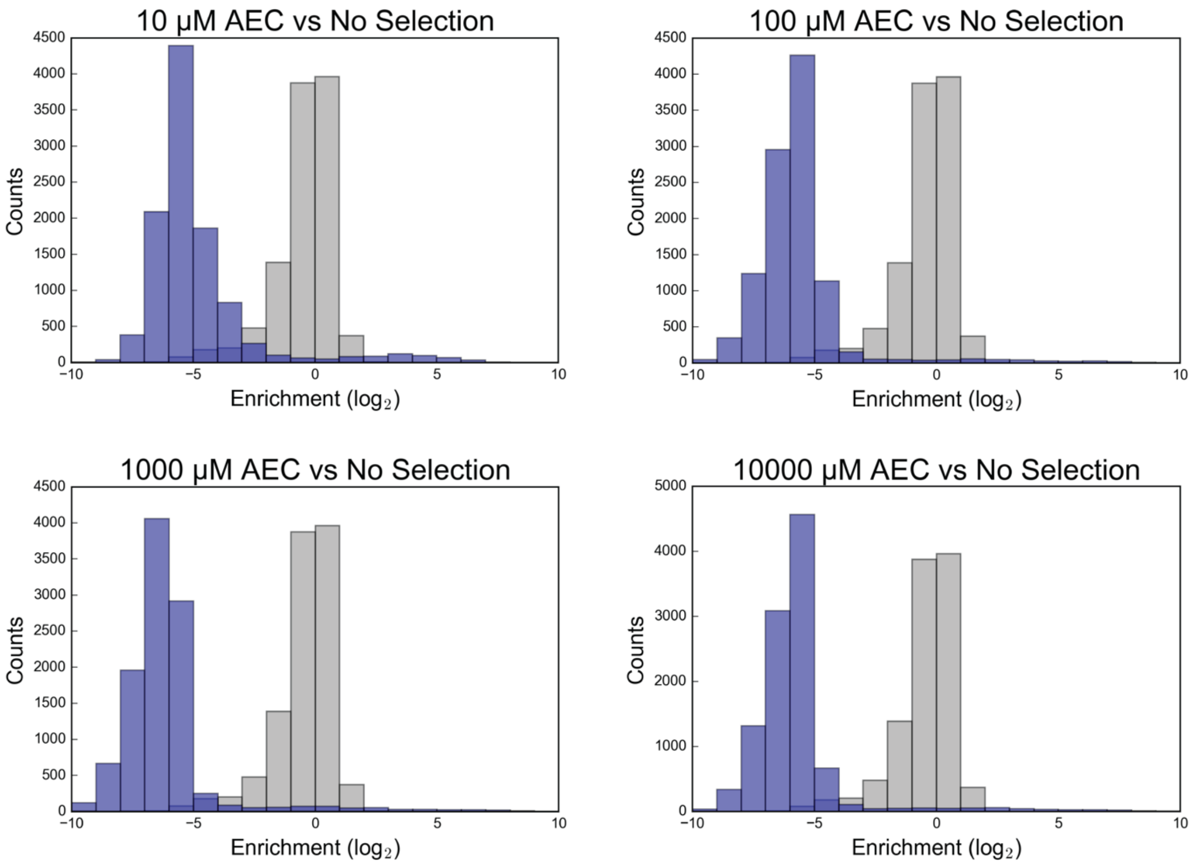

Figure S1. Histogram of log2 enrichment scores in the selected (blue) vs no selection (gray) samples. No selection samples were grown for the same amount of time in minimal media containing no AEC.

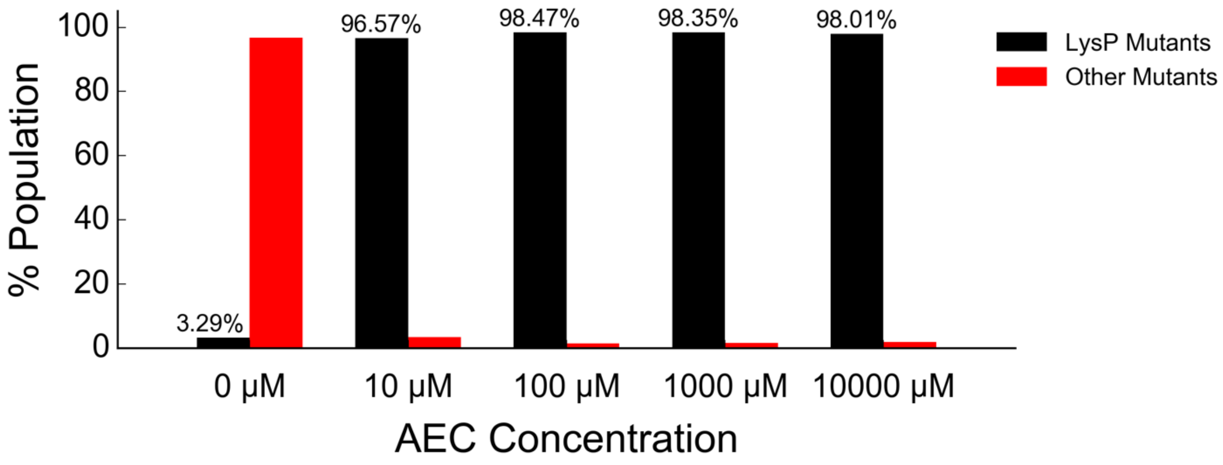

Figure S2. Fraction of lysP mutants in the population across increasing selective pressures. Fractions were calculated from the combined biological replicates.

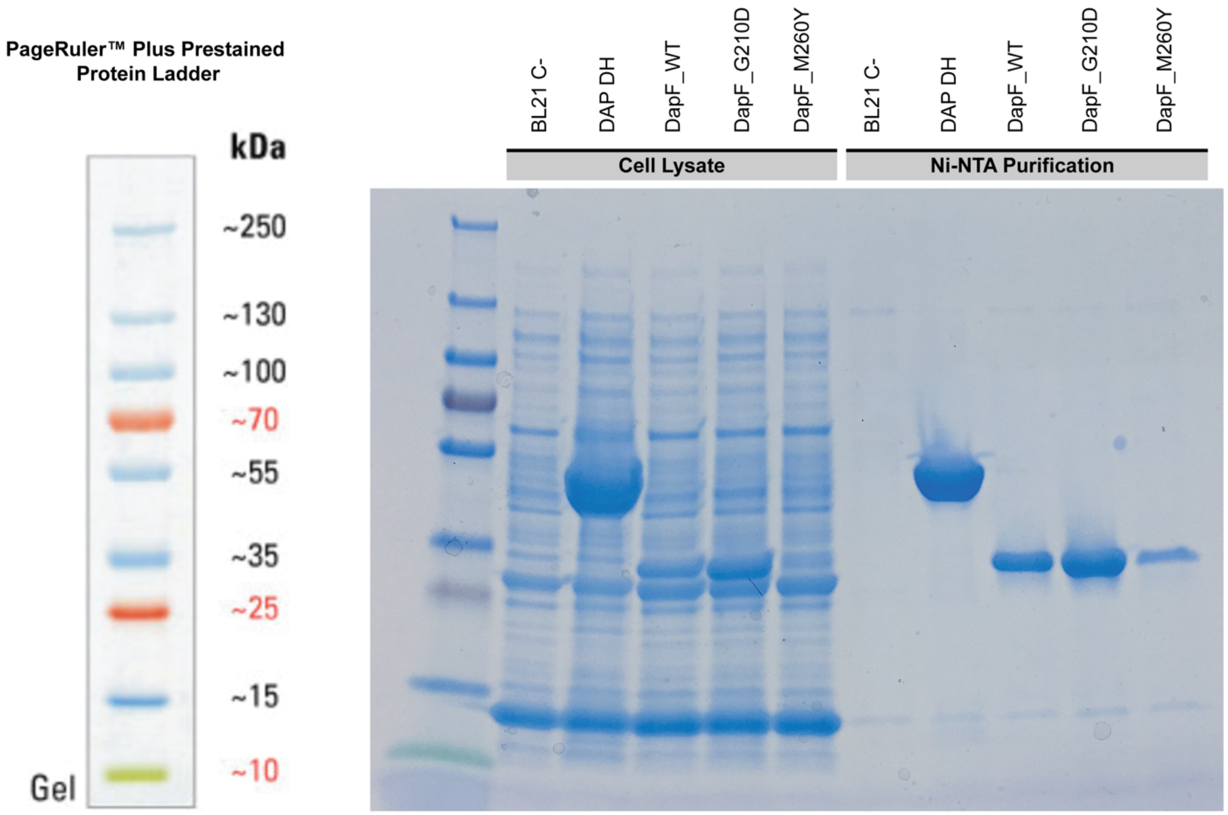

Protein	Length (amino acids)*	Molecular Weight
DAP Dehydrogenase (DAP DH)	283	31.3 kDa
Diaminopimelate epimerase (DapF)	239	36.3 kDa

***Including tag**

Figure S3. Expression and purification of the DapF variants as well as the *Corynebacterium glutamicum* DAP Dehydrogenase. 10uL of induced BL21 cell lysate or the purification eluate were run on a denaturing SDS-PAGE, as described in the methods section.

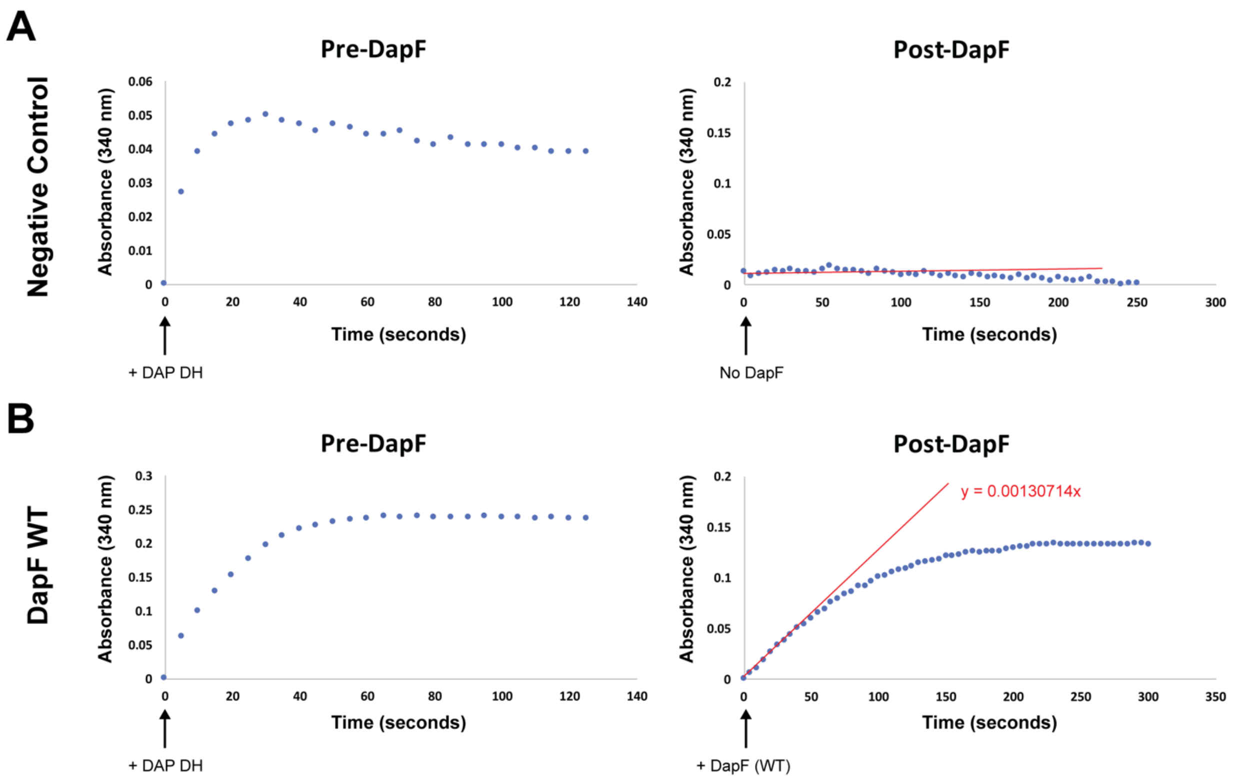

Figure S4. Example of data generated by the *in vitro* DapF kinetic assay. The left panels show the change in absorbance after addition of Dap DH, which is measured until a plateau is attained (i.e. all meso-DAP is depleted). On the right, the change in absorbance is recorded after addition of the purified DapF variant. (a) Negative control, showing no change in absorbance when no DapF is added to the sample. (b) Positive control, showing the change in absorbance after wild-type DapF is added to the mixture. The reaction rate was calculated for a fixed substrate concentration across all samples, using the maximum slope obtained in the change in absorbance measurements.
