# Supplementary material for: Deep scanning lysine metabolism in Escherichia coli
Source: Mol Syst Biol. 2018 Nov 26;14(11):e8371. doi: 10.15252/msb.20188371 (PMC6254735; doi:10.15252/msb.20188371)
Supplement: Supplementary file 2 — Expanded View Figures PDF [file MSB-14-e8371-s002.pdf]

Expanded View Figures

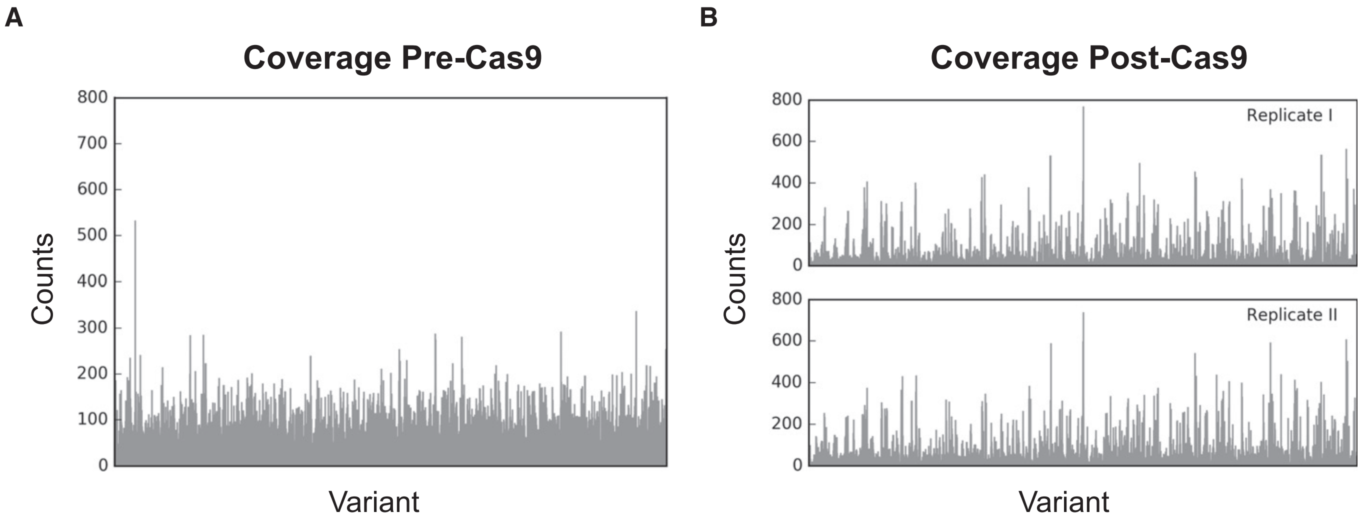

Sample	Libray Coverage
Pre-Cas9 (cloning stage)	99.17%

Sample	Libray Coverage
Replicate I	91.90%
Replicate II	93.79%
Combined	97.07%

Figure EV1. Plasmid library coverage assessed through deep sequencing of the plasmid barcode.

A Coverage of the plasmid library at the cloning stage, before exposure to Cas9.

B Coverage of the plasmid library after exposure to Cas9, in the edited strains. The reported coverage is for pre-selection samples, in the two biological replicates used in this study.

 © 2018 The Authors

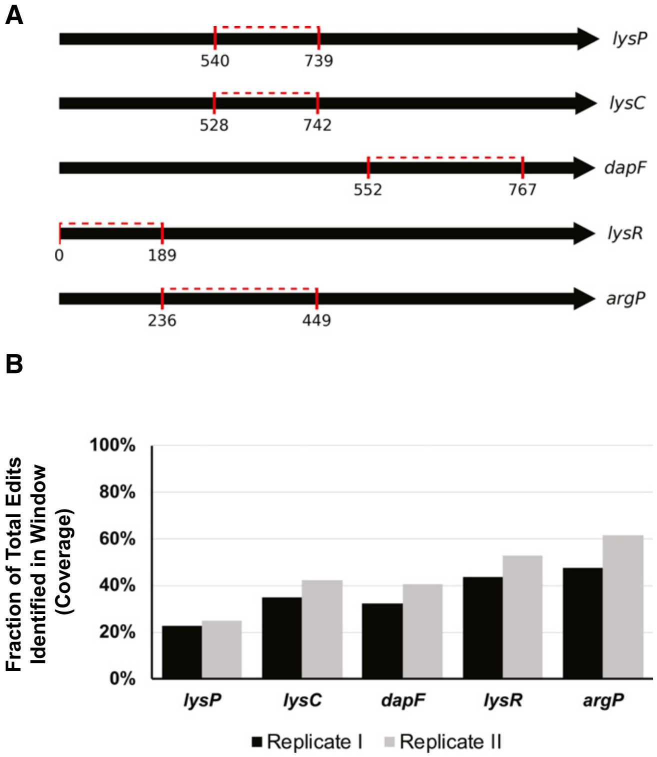

Figure EV2. Deep sequencing of selected genomic regions.

A Regions sequenced in the selected genes. The red marks denote the start position and end positions, with the dashed line highlighting the sequenced genomic region.

B Plot of fraction of designs that could be identified in the sequenced regions (coverage, *y*-axis) for each sequenced gene (*x*-axis).

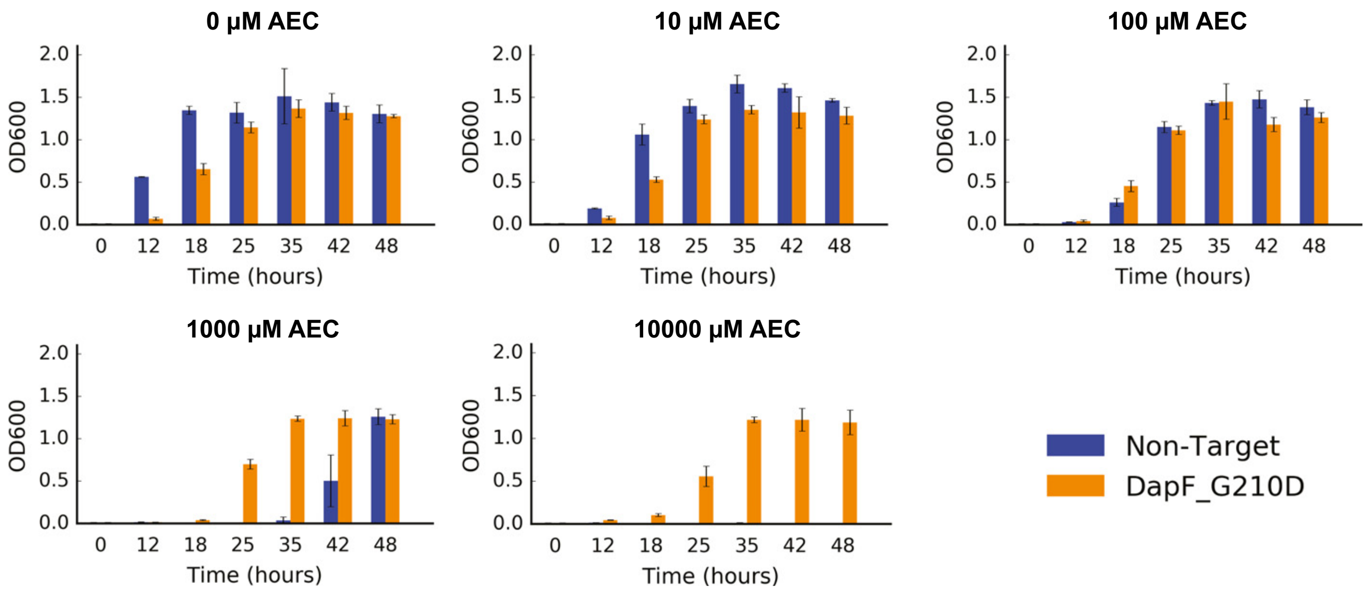

Figure EV3. Growth of the reconstructed DapF G210D mutant compared to wild-type cells transformed with a non-target gRNA ($n = 3$). Error bars show mean value ± SD.

© 2018 The Authors

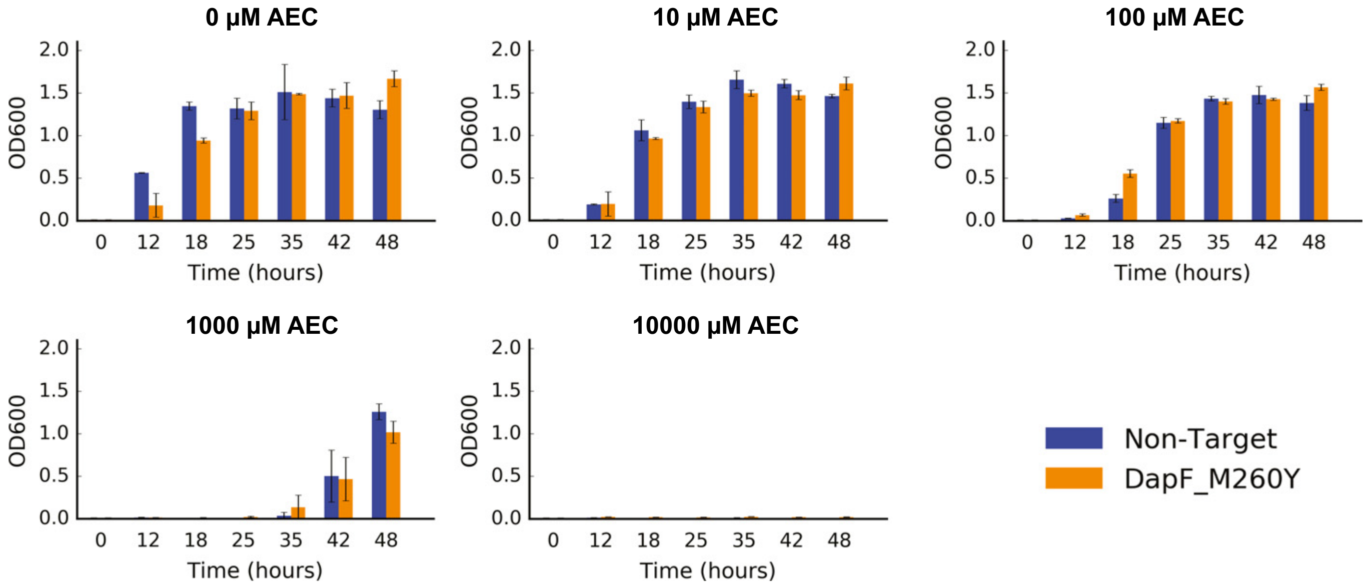

Figure EV4. Growth of the reconstructed DapF M260Y mutant compared to wild-type cells transformed with a non-target gRNA (n = 3). Error bars show mean value ± SD.

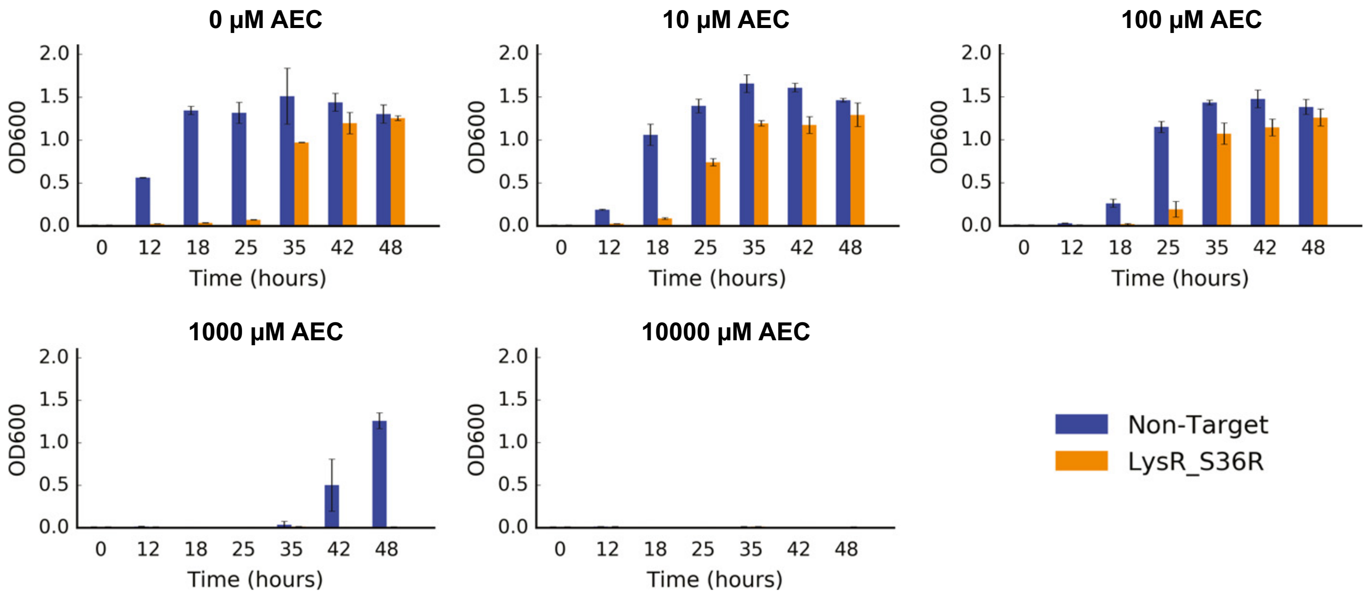

Figure EV5. Growth of the reconstructed LysR S36R mutant (orange) compared to wild-type cells (blue) transformed with a non-target gRNA (n = 3). Error bars show mean value ± SD.

© 2018 The Authors

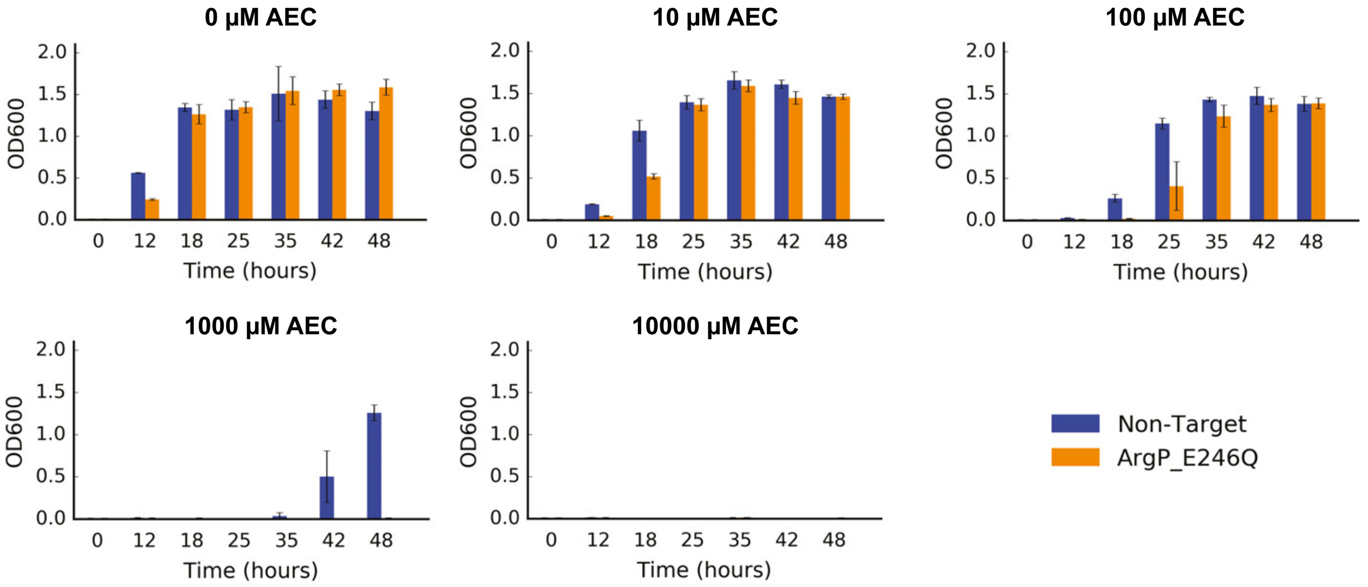

Figure EV6. Growth of the reconstructed ArgP E246Q mutant (orange) compared to wild-type cells (blue) transformed with a non-target gRNA (*n* = 3). Error bars show mean value ± SD.
